# Approach to the Pan-brachial Plexus Injury: Variation in Surgical Strategies among Surgeons

**DOI:** 10.1097/GOX.0000000000003267

**Published:** 2020-11-24

**Authors:** Steven T. Lanier, J. Ryan Hill, Aimee S. James, Liz Rolf, David M. Brogan, Christopher J. Dy

**Affiliations:** From the * Department of Orthopedic Surgery, Washington University School of Medicine, St. Louis, Mo.; † Department of Surgery, Division of Public Health Services, Washington University School of Medicine, St. Louis, Mo.

## Abstract

**Background:**

Treatment of pan-brachial plexus injuries has evolved significantly over the past 2 decades, with refinement and introduction of new surgical techniques, particularly free functional muscle transfer. The extent to which contemporary brachial plexus surgeons utilize various techniques as part of their treatment algorithm for pan-plexus injuries and the rationale underlying these choices remain largely unknown.

**Methods:**

A case scenario was posed to 12 brachial plexus surgeons during semi-structured qualitative interviews. The case involved a young patient presenting 6 weeks after a pan-plexus injury from a motorcycle accident. Surgeons were asked to formulate a treatment plan. Inductive thematic analysis was used to identify commonalities and variation in approach to treatment.

**Results:**

For shoulder function, the majority of surgeons would graft from a viable C5 nerve root, if possible, though the chosen target varied. Two-thirds of the surgeons would address elbow flexion with nerve transfers, though half would combine this with a free functional muscle transfer to increase elbow flexion strength. Free functional muscle transfer was the technique of choice to restore finger flexion. Finger extension, intrinsic function, and sensation were not prioritized.

**Conclusions:**

Our study sheds light on current trends in the approach to pan-plexus injuries in the U.S. and identifies areas of variability that would benefit from future study. The optimal shoulder target and the role for grafting to the MCN for elbow flexion merit further investigation. The role of FFMT plays an increasingly prominent role in treatment algorithms.

##  Introduction

Complete brachial plexus injuries (BPI) are functionally devastating injuries. While there is growing interest in use of nerve transfers and free functional muscle transfer, substantial variability in reconstructive strategies has been noted.^1^ In 2004, Belzberg and colleagues surveyed an international group of experienced peripheral nerve surgeons on the treatment of brachial plexus injuries and found that surgeons employed a variety of techniques to address pan-plexus injuries.^2^ Nonetheless, consensus emerged around certain strategies, including the use of spinal accessory to suprascapular nerve transfer for shoulder abduction and intercostal musculocutaneous nerve transfers for elbow flexion.

The field of brachial plexus surgery has continued to advance since Belzberg’s report was published 16 years ago. One notable change has been the more widespread adoption of free functional muscle transfer since its introduction by Doi for brachial plexus reconstruction in 1997.^3^ The extent to which contemporary brachial plexus surgeons utilize various techniques as part of their treatment algorithm for pan-plexus injuries and the rationale underlying these choices remains largely unknown. An understanding of current areas of consensus and disagreement among surgeons is important to guide future research efforts and to shed light on experiences at various centers. We utilized semi-structured interviews and a hypothetical case example to investigate current areas of consensus and variation in approach to the treatment of pan-plexus among a diverse group of BPI surgeons from centers across the United States.

## Methods

Following approval from our institutional review board, we conducted semi-structured interviews with surgeons who treat patients with BPI in the United States. Purposive sampling was utilized to recruit surgeons based on experience (more or less than 10 years in practice) and training background (plastic surgery or orthopedic surgery). Participants were recruited in person at professional meetings (annual meetings of the American Society of Peripheral Nerve or American Academy of Orthopaedic Surgeons) by the senior author, who is a BPI surgeon with training in qualitative research methods and interview techniques. In-person interviews were conducted for 9 participants, while telephone interviews were conducted for the remaining 3 participants due to scheduling constraints.

The senior author developed the semi-structured interview guide with guidance from a health psychologist with expertise in qualitative research methods, and it was reviewed by 2 additional BPI surgeons. Each participant provided informed consent and completed a demographic survey before the interview. During the interview, participants were asked about their training experiences, interest in BPI treatment, and other influences on their surgical decision-making processes. As part of this process, interviewees were presented the following case vignettes and asked to propose and discuss their management strategy: “A 14-year old was involved in a motor vehicle accident and presents 6 weeks later with signs of a pan-plexus injury, including a flail limb and Horner’s syndrome. Imaging study of choice indicates pseudomeningoceles at C6-T1. Assume that the patient’s recovery has reached a plateau.” Each participant was interviewed individually, with the exception of 1 pair of participants, who work together at 1 center and were interviewed together. All interviews were audio-recorded and transcribed verbatim by a professional transcription company. After the interview, transcriptions were reviewed in full to verify accuracy, and the documents were uploaded into NVivo 12 (QSR International; Victoria, Australia) to facilitate analysis.

Collaborative, iterative methodology was utilized to conduct thematic analyses, including inductive and deductive coding approaches. A preliminary codebook was developed following a review of unique sub-sets of interviews by 4 researchers. This was then refined through discussion and iterative revision to a final codebook that was utilized by 2 researchers to independently code each transcript. Any discrepancies between the coders on individual transcripts were resolved by an independent member of the research team. During interim analyses and following completion of coding, group discussion was used to collate the codes into themes. Surgical strategies proposed by each individual surgeon to manage the pan-plexus patient were analyzed to characterize the spectrum of proposed surgical intervention as well as to determine areas of consensus and variation. Following interim and final analyses of the interview data and subsequent group discussion, the study team determined that data saturation had been achieved (no new themes we emerging from the interview data). No additional interviews were conducted.

## Results

A total of 12 surgeons were interviewed: 9 orthopaedic surgeons and 3 plastic surgeons. At the time of interview, half of the surgeons had been in practice for 5–10 years, a fourth between 11 and 15 years, and a fourth >15 years (range: 5–19 years; median: 9 years). Three surgeons perform >20 reconstructions annually, 3 surgeons perform 11–20 reconstructions, 5 surgeons perform 6–10 reconstructions, and 1 surgeon performs 5 reconstructions per year. All 12 interviewees underwent hand and upper extremity fellowship training at unique, geographically diverse academic institutions and currently practice at 11 different institutions across the United States. Additional information is not provided due to privacy concerns. Individual surgeon comprehensive plans and insight into rationale are displayed in Table 1. In response to the pan-plexus case vignette presented to them, surgeons prioritized elbow flexion, shoulder abduction, and finger flexion as target functions for surgical intervention, in that order. The majority of surgeons (10 of 12) would explore the brachial plexus. Of those that specified timing, 3–4 months after injury was the most common time for initial intervention.

**Table 1.** Comprehensive Plans by Surgeon

Surgeon	Targeted Function	Proposed Surgical Plan	Timing	Comments and Rationale
1	Shoulder abduction and external rotation	Explore plexus: If C5 → graft (target not specified)	1^st^ stage	“I think it’s reasonable [to explore the neck]…you have a significant source of axons and that would allow me then to use spinal accessory to power triceps.”
		If no C5 → SAN to SSN		
	Elbow flexion	Intercostal to biceps and brachialis nn.		
	Elbow extension	+ Intercostal powered FFMT		
	Finger flexion	SAN to triceps (if available root to graft for shoulder)		“SAN to SSN is the best transfer we have for shoulder function of some sort.”
	Wrist	FFMT tendon woven into FDP/FPL	Late	“You can, I think, debate about whether or not you do two free gracilis. I had previously been more apt to do that, but after some practical experience, I think it’s difficult for patients to tolerate two big surgeries like that.”
2	Shoulder abduction and external rotation	No plexus exploration		“[I] would leave spinal accessory on the board for the lower trap transfer down the line.”
		Intercostal to axillary n. transfer	1^st^ stage	
		+ Ipsilateral lower trapezius tendon transfer	2^nd^ stage	
	Elbow flexion	Intercostal to MCN	1^st^ stage	
		+Intercostal powered FFMT		
	Elbow extension	Levator scapulae n. to triceps transfer via nerve graft	1^st^ stage	“Those long nerve grafts from the small nerves in somebody this young—and you got it that early—will probably work…If we can get his elbow extending and bending, and some wrist back, and some digital closure, it’s the best shot.”
	Finger flexion	FFMT tendon woven into FDP/FPL	1^st^ stage	
	Wrist extension	Intercostal to radial nerve transfer via graft	1^st^ stage	
3	Shoulder external rotation	No plexus exploration		“I would offer surgery, and I would do it sooner because I think that would maximize my potential for recovery.”
		Contralateral lower trap transfer		
	Elbow flexion	Intercostals to biceps	1^st^ stage	“By having redundant elbow flexors I can hopefully achieve at least strong, M4 elbow flexion reliably.”
		SAN powered FFMT (Doi)		
	Finger flexion	Intercostal powered FFMT (Doi)	2^nd^ stage	
	Finger extension	SAN powered FFMT (Doi)	1^st^ stage	“Triceps would be nice, but overall, I think less important. They still have one good limb for overhead activities.”
4	Shoulder abduction	Explore plexus	3–4 mo	“I would wait because we have time. we have a few more months. I would see what’s going on with C5 if she is recovering. I would get another EMG.”
		C5 graft to posterior division of upper trunk		
	Elbow flexion	SAN to MCN via graft	3–4 mo	“I think that a re-innervated MCN and biceps functions considerably better than a FFMT for elbow flexion.”
	Finger flexion	Intercostal powered FFMT	2^nd^ stage	
5	Shoulder abduction and external rotation	Explore plexus:	3 mo	Explore to confirm dx, does not trust MRI.
		If C5 → graft to post. div. upper trunk + SAN to SSN		
		If no C5→ SAN to SSN		
	Elbow flexion	C6 nerve graft to MC (if ruptured not avulsed)	3 mo	“I actually struggle with the triceps as a priority because of gravity.”
	Finger flexion	Intercostal powered FFMT	2^nd^ stage	“Fusing the wrist is something I enter into very carefully because I don’t like to lose tenodesis. If you’ve got functional pinch down the road, you want to be able to have some release.”
6	Shoulder abduction and external rotation	Explore plexus:	4 mo	“What’s going on with C5? I think, at six weeks it’s too early to be able to tell. So, I know that people do go to the OR at this point, but we would typically wait until about four months post-injury before we even explore.”
		If C5 → graft to shoulder (target not specified)		
		If no C5→ late arthrodesis v. c/l lower trap transfer (if needed)		
	Elbow flexion	Intercostal to MC	4 mo	“For us, reanimating the hand is an unrealistic goal, and, so, we don’t try to do that.”
	Elbow extension	SAN to triceps via nerve graft	4 mo	
	Wrist	Arthrodesis	2^nd^ stage	
7 and 8 (partners)	Shoulder abduction and external rotation	Explore plexus:	3 mo	“For somebody who’s 30 or under, SAN to SSN—we tend to not do it in the older patients because we like to save the lower trapezius.”
		If C5 → C5 to axillary, SAN to SSN		
		If no C5→ SAN to SSN, possible late arthrodesis		
	Elbow flexion	Intercostals to MC	3 mo	“A couple times on these we’ve also done intercostals to radial trying to get triceps, but generally we just go to biceps.”
	Finger flexion	Intercostal powered FFMT (2^nd^ stage)	2^nd^ stage	“Not on a 14-year-old. If she was a baby we would [address the hand with nerve transfers].”
9	Shoulder abduction and external rotation	Explore plexus: C5 graft to SSN	Not specified	“If I’m doing intercostal nerve transfers, I’ll take the sensory component and graft into the median nerve. In a 14-year-old it probably is worth doing.”
	Elbow flexion	Intercostal to MC	2^nd^ stage	
		+FFMT if needed		
	Sensory	Intercostal to median n.		
10	Shoulder abduction and external rotation	+/- Explore plexus:	Not specified	“I’d look at [C5] electrodiagnostically first, and I’d get MR neurography. If it looks like he’s got a stretch injury then I probably would at least perform a neurolysis… I think the functional test that you do will tell you more than staring at it through the surgical wound.”
		If C5 intact → C5 neurolysis		
		If no C5 → SAN to SSN		
	Elbow flexion	Intercostal to biceps n.	Late	
	Finger flexion	possible FFMT		
	Intrinsic hand and sensation	Intercostal to ulnar n.		
11	Shoulder abduction and external rotation	Explore plexus:	Not specified	“I would look for a C-5 or a C-6 that might be attached…I talk to the pathologist about doing an intra-op fresh-frozen biopsy of the nerve root to assess architecture. If I thought I had a nerve root that was graft-able, that would open up some options for me.”
		If C5 → graft to axillary		
		If no C5→ c/l lower trap tfr. v. arthrodesis	Late	
	Elbow flexion	SAN powered FFMT	Not specified	
12	Shoulder abduction and external rotation	Explore plexus: If C5 → graft to axillary + SAN to SSN If no C5→ intercostals to MC + SAN to SSN	Not specified	“Someone with this type of injury, we can’t do much [for the hand]. If you do a free muscle flap, he still doesn’t have sensation.”
	Elbow flexion	C5 cable graft to MC fascicles of lateral cord	2^nd^ stage	
	Finger flexion	Intercostal powered FFMT (if needed)		

FFMT, free functional muscle transfer; MCN, musculocutaneous nerve; SAN, spinal accessory nerve; SSN, suprascapular nerve.

### Shoulder

To restore shoulder function, the majority of surgeons would graft from a viable C5 nerve root, if possible (9/12, 75%, Table 2). Many surgeons described the availability of the C5 nerve root as an important decision-making factor in their reconstructive plan.

**Table 2.** Shoulder Strategies

Targeted Function	Solutions Proposed by Surgeon Interviewees	No. Surgeons
Shoulder abduction and external rotation	Graft to axillary nerve	1
	Graft to SSN	1
If C5 available	Graft (target not specified)	2
	Graft to posterior division of upper trunk	1
	Graft to post. div. upper trunk + SAN to SSN transfer	1
	Graft to axillary + SAN to SSN	3
	C5 neurolysis	1
If C5 not available	SAN to SSN transfer	6
	c/l lower trapezius tendon transfer v. arthrodesis	2
	No contingency plan stated	2
C5 independent strategy	Intercostal to axillary n. transfer + ipsilateral lower trapezius tendon transfer (2^nd^ stage)	1
	Contralateral lower trapezius tendon transfer	1

SAN, spinal accessory nerve; SSN, suprascapular nerve.

*“I think it’s reasonable to see if there’s anything there. You potentially you have a significant source of axons, and that would allow me then to probably use spinal accessory to power triceps. So, it would change what I would do and change the patient’s overall functional recovery. I think it’s worth the extra, potentially, two to three hours of digging around in the neck.”* (Surgeon 1)

*“If you have C5, that could be a game-changer. But if all you have are intercostals and spinal accessory, then you can try to re-innervate two things…you can use the contralateral lower-third trapezius and get three things.”* (Surgeon 6)

However, the chosen target for a C5 nerve graft varied. Recipients included the suprascapular nerve (SSN, n = 1), posterior division of upper trunk (PDUT, n = 2), and axillary nerve (n = 4). Two surgeons did not specify the target for a C5 graft. Four surgeons combined C5 grafting with an SAN-to-SSN transfer. Among those who did not combine C5 grafting with an SAN-to-SSN transfer, reasons cited included saving the SAN as a donor for other functions and a desire to use the ipsilateral trapezius for later tendon transfer. For a scenario in which C5 is not available, 6 of the 10 surgeons who chose to explore the brachial plexus would perform an SAN-to-SSN transfer. Two surgeons would treat the shoulder with a lower trapezius tendon transfer or arthrodesis rather than nerve transfers. Two surgeons did not detail a contingency plan for a non-viable C5 root. The two surgeons who elected not to explore the brachial plexus would treat the shoulder with an ipsilateral or contralateral lower trapezius transfer.

### Elbow

To address elbow function, most surgeons focused on restoring elbow flexion and would utilize nerve transfers, with substantial agreement on the use of intercostal nerves (ICN) as donors (8/12, 66%, Table 3). The proper musculocutaneous nerve (MCN) was the most common recipient chosen for this transfer (7/8, 88%), though 1 surgeon chose to perform ICN transfers directly to the nerve to biceps (distal to the proper MCN). Half of the surgeons who planned ICN-to-MCN transfer would supplement this with a free functional muscle transfer (FFMT, 4/8, 50%). One surgeon elected to transfer the SAN extended by a nerve graft to the MCN. A minority of surgeons would attempt to restore elbow flexion with grafting from C5 or C6 nerve roots to the MCN (2/12, 17%). Only 3 surgeons addressed elbow extension. Two would utilize a graft from the SAN to triceps motor branch, and 1 a graft from the dorsal scapular nerve.

**Table 3.** Elbow Strategies

Targeted Function	Solutions Proposed by Surgeon Interviewees	No. Surgeons
Elbow flexion	Intercostal to musculocutaneous nerve transfer	3
	Intercostal to musculocutaneous nerve transfer	3
	+ Intercostal powered FFMT	
	Intercostals to musculocutaneous nerve transfer	1
	+ SAN powered FFMT	
	+Intercostal powered FFMT (Doi)	
	Intercostal to biceps nerve transfer	1
	SAN powered FFMT	1
	SAN to musculocutaneous nerve transfer w/nerve graft	1
	C5 nerve graft to lateral cord (musculocutaneous fibers)	1
	C6 nerve graft to musculocutaneous	1
Elbow extension	SAN to triceps nerve transfer	2
	Levator scapulae to triceps nerve transfer	1

FFMT, free functional muscle transfer; SAN, spinal accessory nerve; SSN, suprascapular nerve.

*“The triceps would be nice to have, but overall, I think less important. They still have one good limb for overhand activities and presumably they’re not going to be in a wheelchair and need a triceps for transfers.”* (Surgeon 3)

### Hand Function and Sensation

Fewer surgeons voiced strategies to address hand function in the context of a pan-plexus injury (Table 4). Slightly more than half would attempt to restore finger flexion (7/12, 58%), all via an FFMT. Five of the 7 (72%) would utilize an FFMT primarily for finger flexion, whereas 2 surgeons who utilized an FFMT for elbow flexion would weave this distally into the finger flexors. Only 1 surgeon addressed finger extension, which was via the Doi method of double FFMT.

**Table 4.** Hand Strategies

Targeted Function	Solutions Proposed by Surgeon Interviewees	No. Surgeons
Finger flexion	Intercostal powered FFMT	5
	Tendon graft extension of FFMT for elbow	2
Finger extension	SAN powered FFMT (Doi)	1
Hand intrinsic function and sensation	Intercostal to ulnar nerve transfer	1
Thenar and sensation	Intercostal to median nerve transfer	1

FFMT, free functional muscle transfer; SAN, spinal accessory nerve.

*“I think if I were 14, and I wanted to have a maximum chance for recovery in in the future, I think doing an early double Doi would give me the most options. And I think that a younger patient does better with that operation. By having redundant elbow flexors I can hopefully achieve at least strong, M4 elbow flexion reliably and hopefully achieve some grasp and release as well.”* (Surgeon 3)

One surgeon would address intrinsic function and sensation, utilizing an ICN to ulnar nerve transfer, and 1 surgeon would address thenar function and sensation via an ICN to median nerve transfer.

*“If I’m doing intercostal nerve transfers, I’ll take the sensory component and graft into the median nerve.In a 14-year-old, it probably is worth doing.”* (Surgeon 9)

Definitions of success were tempered and focused on painless shoulder stability and the ability to bend the elbow to assist with dressing, hygiene, and feeding (Table 5).

**Table 5.** Definitions of Success

Surgeon 1	“I would tell them, right off the bat, that there is no way we can make their arm anywhere near normal and that our goal is to give them some form of a helper hand. I think it’s realistic to hope that there is some-some form of shoulder function, some form of elbow flexion, and, perhaps, uh, some sort of rudimentary grasp. They won’t have independent finger or thumb flexion. They won’t have any sort of intrinsics. It’s a relatively weak grasp, but it’s a grasp of some sort.”
	“They have some strength, but can’t lift anything heavy with that hand. It’s a helper hand. It’s hard for them to pick up something on their own. They usually have to place it into the hand because of the mechanics of the grasp.”
Surgeon 2	“If they get something back to the hand it’s gross motor control…simple grasp, but not fine, dexterous activities. Some people have called it a dumb hand…it doesn’t have finesse or any fine motor.”
	“[Patients] have said that the surgeries gave them some shoulder stability and a little bit of motion, and bending the elbow has helped the arm to feel part of their body when they’re ambulating. If it’s not connected to the body, ambulation is thrown off. It doesn’t hurt as much, because it’s actually not just dragging, and they can bend their elbow to get it out of the way of things.”
Surgeon 3	“[T]he more we try to achieve in general, you know, if we’re doing a big double Doi, I think it’s less certain that you’re going to achieve those goals. With other operations, if you’re only trying to achieve one or two major functions, I think it’s more reliable, with less upfront cost.”
	“I’ve been happy with the amount of elbow flexion I’ve been able to gain. [T]he grasp and release I feel is very limited, but to that end, um, you know, cortically the patients are able to signal grasp and release actively.”
Surgeon 6	“We try to get elbow flexion, elbow extension, recognizing that we may be able to get some mobility out of the shoulder with [trapezius transfer]…or they can have the shoulder fused. If they get the elbow back, then we’ll fuse the rest. And, I think that, to me, is not a bad outcome. A little bit will also depend on whether they have some scapular control… a lot of these patients seem to have some scapular control. I think, to me, that’s the most straightforward approach—it takes a long time, but it’s the most predictable, low-cost approach you can get to achieve a functional arm with somebody that has, really, basically nothing.”
Surgeons 7 and 8	“We tell them that if you get to the point where you can control your shoulder and you can bend your elbow up to your mouth, that is a home run for this.”
Surgeon 9	“I think, you know, they can position their arm in space. If their elbow flexion is strong enough, they can hold something. It helps them a little bit for activities of daily living like eating and even getting dressed. But that’s kind of the extent of it often.”
Surgeon 10	“I tell them hygiene is my goal for them, and feeding themselves…anything after that they’re going to have to develop some level of ingenuity to accomplish, and work with the therapist.”
	“If we can get your hand to your mouth and your hand to your butt, you can zip or unzip your pants, that that is a reasonable outcome to try to shoot for.”
Surgeon 12	“We don’t expect him to have hand function, though patients find this to be a functional helper arm. Because they have elbow flexion they can hold a lot of things with the elbow.
	They can control the shoulder, so they can wash without holding their arm.”

*“They have some strength, [but] they can’t lift anything heavy with that hand. It’s a helper hand and it’s hard for them to pick up something on their own. They usually have to place it into the hand because of the mechanics of that grasp. And I think it takes a long time. And so, I think patients, when they finally get something, it’s a limited use.*” (Surgeon 1)

Influence of mentorship, personal experience, and patient age were factors in choosing operative strategies that were highlighted by multiple interviewees.

*“One thing I learned in my travels is that [pan plexus cases] are extremely heterogeneous in how they’re treated. I mean, highly varied and not great results by anybody… okay results for some people. It’s just a combination of my experience, what I hear from other people, and what I’ve seen.”* (Surgeon 2)

*“My philosophy very quickly shifted after a few really long cases with suboptimal recovery to how do I balance the effort versus the predictability.”* (Surgeon 6)

## Discussion

Our study sheds light on current trends in the approach to pan-plexus injuries among a varied group of expert surgeons from centers across the United States. Surgeons agreed on prioritizing shoulder function, elbow flexion, and grasp. This mirrors a broader consensus in the literature underpinned by the rationale that shoulder stability and elbow flexion are pre-requisites to position the hand in space for useful function.^4^ The highest degree of consensus existed around restoring elbow flexion and the use of ICN-to-MCN transfers to achieve this, which reflects this transfer’s long history and track-record of success.^5^ The use of extraplexal donors other than the ICN or SAN (eg, phrenic nerve or contralateral C7) has been eschewed by most surgeons in North America due to concerns for safety and morbidity, and this was reflected in the responses we received.

Interestingly, we found that more than half of surgeons would perform FFMT as a primary strategy to restore elbow flexion, either alone or in combination with nerve transfers. Our findings indicate increased utilization of this technique and may represent evolving thought with respect to indications for FFMT as greater experience is accumulated. A recent systematic review of 19 articles and 364 patients, the majority with pan-plexus injury, found that 87% achieved M ≥ 3 and 65% achieved M ≥ 4 elbow flexion following FFMT, with a mean total elbow flexion of 58–107 degrees and a 27-point improvement in DASH scores.^6^ Furthermore, a large cohort study found FFMT alone to be equally effective to FFMT combined with ICN transfer, concluding that the latter may not even be needed.^7^ Two surgeons chose grafting from a viable root directly to the MCN for restoration of elbow flexion, a strategy that has been less commonly utilized but advocated by some authors with comparable success to FFMT in small series.^8^ Future studies can help better define the role of these newer strategies to restore elbow flexion in pan-plexus patients.

Shoulder stability and function were predominantly addressed with re-innervation strategies. Nearly all surgeons who explored the plexus would perform nerve grafting of a viable C5 nerve root, citing the desire to utilize any available axons. Up to 80% of pan-plexus injuries may have at least one viable nerve root for grafting, and 2 systematic reviews indicate an equal likelihood of attaining functional M ≥ 3 shoulder abduction with nerve grafting or single nerve transfer.^9–11^ Four surgeons interviewed combined nerve grafting with an SAN-to-SSN transfer, which has been shown to result in superior outcomes to nerve transfer alone for upper trunk brachial plexus injuries.^12^ Choice of a distal target for a C5 graft varied substantially, including the PDUT, SSN, or axillary nerve. This variation evidences a need for future comparative data to guide this choice.

In the absence of a viable C5 nerve root, there was a relative consensus on the use of SAN-to-SSN transfer to target shoulder function, after which 70% of patients attained M ≥ 3 and 35% M ≥ 4 shoulder abduction with an average of 45 degree of external rotation in a large meta-analysis of upper trunk injuries.^13^ While only 3 surgeons discussed trapezius tendon transfer and shoulder arthrodesis as options, consideration should be given to these strategies before sacrifice of the SAN. Trapezius tendon transfer has been shown to significantly improve external rotation and modestly improve shoulder abduction to a mean of 50°, while long-term outcomes of shoulder arthrodesis after pan-plexus injury compare favorably to nerve transfer in terms of range of motion, strength, DASH scores, and patient satisfaction.^14–17^

Eight of the 12 surgeons interviewed would attempt to restore finger flexion, and intercostal powered FFMT was the unanimous strategy among those who did. FFMT can successfully restore finger flexion to achieve either hook grip or weak grasp, depending on the addition of tendon transfers to address intrinsic minus clawing.^4,18,19^ Long-term results by Doi and colleagues found that slightly greater than 50% of patients achieve controlled prehension and routinely use the reconstructed extremity.^20,21^ No surgeons interviewed elected to utilize a nerve transfer based strategy to achieve finger flexion, which is reflective of generally poor outcomes reported with attempts to directly reinnervate the median or ulnar nerves.^22–31^ However, ICN to median and ulnar nerve transfers can attain return of S2 protective sensation in 65% of patients undergoing ICN to median or ulnar nerve transfer, which allowed the patient to identify the presence of an object touching his or her hand and identify hot and cold.^22,32^ Prior authors have stressed the importance of protective sensation to useful hand function following FFMT to restore grasp.^19,22^ Future studies geared toward understanding limitations in the way pan-plexus patients are using their reconstructed hand will help define the role of sensory transfers.

Our study has several limitations. Because surgeon case plans were elicited as part of an interview without prior knowledge of the case, further reflection may have resulted in changes to the voiced surgical plans. Although we included both orthopedic and plastic surgeons, we did not include any neurosurgeons who may have different perspectives on treatment, which limits the generalizability of our results across all US centers. We did not interview surgeons who practice outside the United States, which limits the applicability of our findings but serves an opportunity for future investigation. Nonetheless, our sampling strategy allowed us to interview surgeons of varying experience and training backgrounds, likely capturing much of the variability in treatment philosophies.

## Conclusions

Our study sheds light on current trends in the approach to pan-plexus injuries in the United States and identifies areas of variability that would benefit from future study. While there was general agreement on C5 grafting when possible, the optimal shoulder target and the role for grafting to the MCN for elbow flexion merit further investigation. The role of FFMT continues to evolve, and further studies may better define the role of ICN-to-MCN transfer and FFMT to restore elbow flexion. The areas of consensus and variation in our interviews mirror the existing literature on treatment of pan-plexus injury and, in important respects, are similar to the findings of Belzberg and colleagues over 15 years ago, with the exception of an increased role for FFMT.^3^ Sentiments expressed by surgeons interviewed reinforce the devastating nature of pan plexus injuries and the fact that, despite best efforts, meaningful recovery is difficult to achieve with current surgical techniques. It is notable that there was no unanimously chosen strategy, demonstrating the variation in opinions that remains for pan-plexus injuries.
